# The MAPK Response to Virus Infection Is Modified by Probenecid

**DOI:** 10.3390/cimb47040246

**Published:** 2025-04-02

**Authors:** Les P. Jones, David E. Martin, Ralph A. Tripp

**Affiliations:** 1 Department of Infectious Diseases, University of Georgia, Athens, GA 30605, USA; lj66@uga.edu; 2 TrippBio, Inc., Jacksonville, FL 32256, USA; davidmartin@trippbio.com

**Keywords:** virus, respiratory syncytial virus (RSV), extracellular signal-regulated kinases (ERKs), c-Jun N-terminal kinases (JNKs), MAPK, mitogen-activated protein kinase p38 (p38), cell signaling, innate immunity, probenecid

## Abstract

Respiratory viruses such as respiratory syncytial virus (RSV) annually cause respiratory illness, which may result in substantial disease and mortality in susceptible individuals. Viruses exploit host cell machinery for replication, which engages the mitogen-activated protein kinases (MAPK) pathway. The MAPK signaling pathways are triggered by pattern recognition receptors that recognize the pathogen, infection, or external stimuli, leading to the induction and regulation of immunity and inflammation. Probenecid, used to improve renal function by inhibiting the tubular reabsorption of uric acid, has been shown to have therapeutic efficacy in reducing inflammation and blocking viral replication by inhibiting components of the MAPK pathway that preclude virus replication. This review summarizes key molecular cascades in the host response to virus recognition, infection, and replication and how this can be altered by probenecid treatment.

## 1. Respiratory Viruses

Respiratory viruses cause respiratory tract illnesses and share clinical symptoms and prevention strategies. Common respiratory viruses include influenza, severe acute respiratory syndrome coronavirus 2 (SARS-CoV-2), respiratory syncytial virus (RSV), human metapneumovirus (HMPV), parainfluenza, and adenoviruses [1]. In temperate climates, respiratory viruses may cause seasonal epidemics. Respiratory pathogens such as seasonal influenza, RSV, and HMPV circulate in many countries from winter to spring. Viruses like influenza can cause seasonal infections, carry a high disease burden, and cause hundreds of thousands of hospitalizations and deaths [2]. Different groups of people are at higher risk for increased morbidity and mortality from respiratory disease, specifically older adults, people with underlying health conditions, young children with developing immune systems, people with weakened immune systems, and people with underlying medical conditions, as well as pregnant and recently pregnant people [3,4,5,6]. Several core disease prevention strategies, such as immunization for influenza, SARS-CoV-2, and RSV, are available, as well as drug treatment options, as vaccination or treatment can reduce severe disease, hospitalization, and death.

## 2. RSV Infection, RSV Glycoproteins, and MAPK Induction

RSV is a common cause of infant hospitalization, may cause reinfections throughout life, and is a notable pathogen affecting all age groups. Infection rates are higher in winter, causing bronchiolitis in infants, common colds in adults, and pneumonia in the elderly and immunocompromised [7,8]. Globally, RSV causes acute respiratory infection in infants, leading to >60,000 deaths and >3 million hospital admissions/year in children <5 years old [9]. The RSV genome is a negative-sense single-stranded RNA encoding 11 proteins [10]. The RSV F and G surface glycoproteins are key in virus entry, replication, and immune modulation. The F and G glycoproteins interact with the host cell and principally initiate the MAPK cascade. Indeed, when expressed after infection, other RSV proteins can affect the MAPK cascade, but this review focuses on the earliest interactions between the F and G proteins and the outcome of infection. The F protein is the viral fusion protein that interacts with TLR4 and can activate nuclear factor kappa-light-chain-enhancer of activated B cells (NF-κB) to induce proinflammatory cytokines, e.g., tumor necrosis factor-alpha (TNFα), interleukin-6 (IL-6), and interleukin-12 (IL-12) [11]. The G protein is the attachment protein that binds to ciliated respiratory epithelial cells primarily through the G protein region CX3C and CX3CR1 (fractalkine receptor) interaction [12]. The G protein activates innate immunity by interacting with Toll-like receptor-2 (TLR2) [13]. Additionally, the G protein encodes a central conserved region containing a CX3C motif that functions as a fractalkine mimic [14]. The disruption of the G protein CX3C motif has been shown to affect G protein function in vitro and the severity of RSV disease pathogenesis in vivo [12].

Toll-like receptor (TLR) stimulation by RSV leads to the activation of MAPK signaling pathways and the transcriptional regulation of TLRs, as well as inducing an array of transcription factors, such as Interferon Regulatory Factor 3 (IRF3), Interferon Regulatory Factor 7 (IRF7), NF-κB, c-Jun, and Activator Protein-1 (AP-1), which translocate to the nucleus and initiate the transcription of various proinflammatory cytokines. It has been shown that ERK activity is required for RSV-induced interleukin-8 (IL-8) production in airway epithelial cells, and ERK activity is required for lipopolysaccharide (LPS)-induced TNFα [15]. The inhibition of p38 MAPK impacts the virus–host interaction, and p38 MAPK affects the inflammatory response associated with the expression of proinflammatory molecules [16,17,18]. Interestingly, one study showed that RSV activates p38 MAPK in a Toll-like 4 (TLR4)-mediated manner during the early stage of infection, utilizes p38 MAPK for replication [19], and sequesters the NF-κB p65 subunit in cytoplasmic inclusion bodies to inhibit innate immune signaling [20]. Also, ERK activation and transport of RSV F protein to the plasma membrane has been described [21], and it has been shown that the phosphorylation of the RSV small hydrophobic (SH) protein occurs via a MAPK p38-dependent pathway [22]. Thus, components of the MAPK pathway regulate gene expression and function, and viruses have taken advantage of this pathway to aid their replication and/or preclude antiviral responses. These findings are consistent with the critical role of MAPK in regulating the host response to virus infection and the numerous inflammatory mediators/cytokines expressed in response to RSV infection. Typically, disease pathogenesis following infection is a multifactorial process involving virus replication, innate responses to infection, and aberrant adaptive immune responses that can be linked to modification by RSV proteins. The early onset of RSV disease severity suggests that features affecting innate immunity have an essential role in the disease process, and it is likely that these features are linked to RSV activation of pattern recognition receptors (PRRs)/TLRs [23].

Non-respiratory viruses also affect MAPK signaling. For example, Ebola viruses (EBOV) are hemorrhagic fever viruses spread through direct contact with an infected person’s bodily fluids [24]. It has been shown that EBOV minor matrix protein (VP24), a protein that inhibits the antiviral response, is associated with the nucleocapsid and is critical for producing infectious viral particles [25]. Significantly, EBOV VP24 can block the p38 MAPK pathway, while the EBOV soluble glycoprotein (sGP) can activate the MAPK signaling pathway [26]. EBOVs are highly pathogenic with high human fatality rates. Six distinct EBOV species are known, i.e., Zaire, Sudan, Taï Forest, Bundibugyo, Bombali, and Reston. EBOV sGP contributes to pathogenesis by activating the MAP kinase signaling pathway and increasing its pathogenicity [26].

## 3. MAPK Cascade

Pathogen-associated molecular patterns (PAMPs) are chiefly recognized PRRs, TLRs, and retinoic acid-inducible gene I (RIG-I)-like receptors [27]. Ideally, a host recognizes the pathogen, allowing for an effective immune response. Recognition occurs by signals originating from PRRs (i.e., TLRs, RIG-I-like receptors, and nucleic acid receptors), which initially signal for innate immune response and induce a MAPK cascade. MAPKs are involved in host cell signaling pathways and are known to be activated by virus infection and replication [28]. MAPK cascades are conserved across plants and animals and are essential to immunity [29]. They convert extracellular signals into a wide range of cellular responses. The MAPK pathway regulates host gene expression, cell survival, and apoptosis via protein–protein interactions. MAPKs phosphorylate serine and threonine residues in proteins to convert extracellular signals into cellular responses. The MAPK cascade includes MAP kinase kinase kinases (MAP3Ks), MAP kinase kinases (MAP2Ks), and MAP kinases (MAPKs) [30]. MAP3Ks function as links in signal transmission through distinct protein–protein interactions and the phosphorylation of signal effectors. Among the 24 MAP3Ks identified, different MAP3Ks can phosphorylate the same MAP2K [31]. MAP3Ks serve as hubs integrating cellular responses to offer specificity in functional responses.

**Figure 1:**
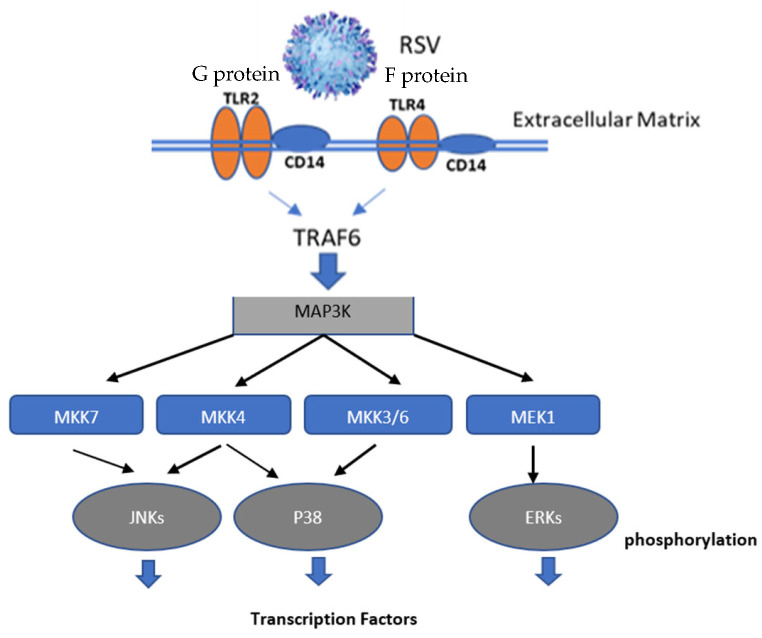
RSV infection induces TLR signaling. TLR2/TLR4 signaling is activated by RSV G (TLR2) and RSV F (TLR4) proteins. TLR stimulation recruits the myeloid differentiation primary response 88 (MyD88) adaptor protein, which interacts with TNF receptor-associated factor 6 (TRAF6), which helps transmit signals between receptors and other proteins to activate MAPKs and induce the translocation of NF-κB and the production of cytokines. CD14, a glycosylphosphatidylinositol (GPI)-linked protein, can chaperone TLR agonists like lipopolysaccharide (LPS) to the TLR4 signaling. Central to the MAPK signaling pathway is a three-tiered kinase module comprising MAP kinase kinase kinase (MAP3K). Upon activation, MAP3K phosphorylates and activates MAP2K, which then phosphorylates and activates MAPK. This sequential activation is a hallmark of the MAPK signaling pathway. There are several MAPK pathways, the most studied being ERK, JNK, and p38 MAP kinases. Different stimuli activate each of these pathways and lead to distinct cellular responses. Mitogen-activated protein kinase kinase 7 (MKK7) is an essential component of the JNK signal transduction pathway activated by proinflammatory cytokines. Mitogen-activated protein kinase kinase 4 (MKK4) directly phosphorylates and activates JNK in response to cellular stresses. Mitogen-activated protein kinase kinase 3/6 (MKK3/6) is the main MAPKK that phosphorylates and activates p38 MAPK. MAP2K1, or MEK1, mediates tyrosine phosphorylation and threonine in ERK1 or ERK2.

MAPK signaling may be activated by virus–host interaction, replication, and associated cellular stresses [32,33,34]. All viruses affect host kinases in regulating virus replication. The MAPK pathway controls host gene expression by phosphorylating serine and threonine residues to convert extracellular signals into cellular responses [35]. ERK, JNK, and p38 kinase are mammals’ three major MAPK pathways [36]. ERK is a key molecule since activated ERK can phosphorylate downstream kinases in the cytoplasm, at the cell membrane, and in the nucleus, thus broadly affecting the signaling cascade. ERK may phosphorylate several transcription factors, such as the ETS proto-oncogene 1 (Ets-1), c-Jun, cellular Myc (cMyc), NFκB, ETS domain transcription factor (Elk1), c-Fos, and Signal Transducer and Activator of Transcription 1 (STAT1) [37]. JNKs are encoded by JNK1, JNK2, and JNK3, where JNK1 and JNK2 are ubiquitously expressed, while JNK3 is limited to the brain, heart, and testis [38]. JNK regulates apoptosis via the modulation of c-Jun/AP1 and transforming growth factor-β (TGFβ) [39]. The p38 MAPK pathway is a central mediator in cellular protein synthesis, modulating macrophages and neutrophils, and mediating T cell differentiation and apoptosis by regulating interferon-gamma (IFNγ) production [40] (Figure 1).

MAPK signaling occurs when a cell confronts stress, such as oxidative stress, ultraviolet (UV) radiation, cytokines, heat shock, DNA damage, or viral infection [35]. In the case of virus interaction, MAPK pathways are actuated by viruses binding to PPRs/TLRs (Figure 1) and by secretory proteins, such as cytokines, that can trigger ERK activation [41]. The MAPK pathway is biphasic in that both early and late phases of infection are linked to virus replication and viral protein expression. Interestingly, different viruses may utilize the MAPK pathway to various extents. For example, the replication of SARS-CoV-2 is thought to be less dependent on MAPK/ERK signaling than the influenza virus, as influenza relies more heavily on the early stages for replication, engaging the transmembrane protease, serine 2 (TMPRSS2)-mediated cell surface entry mechanism [42]. However, both influenza and SARS-CoV-2 replication is inhibited by probenecid [43], emphasizing the importance of the JNK and ERK phosphorylation involving the MAPK pathway.

## 4. MAPK Inhibitors

MAPK signaling pathways are highly conserved across eukaryotes and share a typical structure and function from yeast to humans [44]. For these reasons, it was considered feasible to repurpose MAPK pathway inhibitors to alleviate viral infections. The advantage of targeting host genes is that they are not easy to mutate, and drug resistance rarely occurs during short-period viral treatment. In addition, as almost all oncogenes directly or indirectly lead to the activation of the MAPK pathway, significant efforts have been made to develop drugs to interfere with this pathway, explicitly using MEK inhibitors that target the Ras/Raf/MEK/ERK signaling pathway that involve small GTPases that act as molecular switches relaying signals from cell surface receptors to downstream signaling pathways which inhibit cell proliferation and induce apoptosis [42,45]. Various MAPK inhibitors have been investigated principally for their antineoplastic activity. Regrettably, MAPK inhibitor toxicities are common [46] and MAPK inhibitors are ineffective as treatments because the rapid replication of the viral genome engenders frequent mutations, favoring the generation of drug-resistant variants [47]. Thus, different host-directed MAPK pathway inhibitors are sought to control virus replication and prevent disease pathogenesis.

## 5. Probenecid as a Therapeutic Antiviral Treatment

Probenecid is recognized as a versatile drug in pharmacological therapies, primarily because it blocks active tubular secretion in the kidneys [48]. Probenecid was developed in 1949 to decrease the renal clearance of penicillin. The drug was approved by the FDA in 1951 for treating gout and is considered safe and well-tolerated at doses up to 2000 mg per day [49]. This function affects endogenous substances like uric acid and exogenous substances like penicillin [50]. Its mechanism of action is through the competitive inhibition of organic anion transporters, which are responsible for the excretion of organic agents, such as penicillin. For patients taking probenecid for gout or to help remove uric acid, the amount of uric acid in the kidneys is significantly increased, which may cause kidney stones or other kidney problems in some people. Probenecid is typically used in combination with colchicine, and common side effects include headache, gastrointestinal upset, and hypersensitivity reactions. While known to inhibit specific transporters at the blood–brain barrier (BBB), it does not readily cross the BBB itself. Instead, it blocks efflux transporters like Organic Anion Transporter 1 (OAT1) and 3 (OAT3), potentially increasing the brain’s exposure to certain drugs [51]. Probenecid has emerged as a potent antiviral agent, showing in vitro and in vivo efficacy against multiple respiratory viruses. The antiviral effects of probenecid depend on factors including the host cell type that the virus infects, the virus type, strain, or variant infecting the cell, and whether the drug was used prophylactically or therapeutically. For example, the representative IC_50_ values for several influenza variants are shown for probenecid treatment in the Table 1 below.

Probenecid prophylactic or therapeutic treatment has been shown to inhibit the replication of influenza A and B viruses, SARS-CoV-2 (and variants), RSV (strains A and B), and HMPV (strains A and B) in different human respiratory epithelial cells and mice [43,49,52,53,54,55,56]. A clinical trial involving moderate-to-severe COVID-19 patients confirmed probenecid’s antiviral activity [49]. Given the broad antiviral activity for many virus types, strains, and variants, the antiviral effects of probenecid are likely related to the shared components of the MAPK host cell pathway needed by viruses for their replication. Indeed, probenecid treatment has inhibited JNK and ERK phosphorylation and the downstream phosphorylation of required c-Jun for virus replication, cyclooxygenase-2 (COX-2), and reactive oxygen species (ROS) generation [57,58]. The inhibition of MAPK components by probenecid treatment also has significant anti-inflammatory properties, inhibiting pro-inflammatory cytokines and inflammasome activation. Probenecid treatment reduces the proinflammatory response, related proinflammatory cytokines, and inflammation [53,59,60,61,62]. In an earlier study, probenecid was shown to reduce activated caspase-1 and the activation of the Nod-like Receptor Protein 1 (NLRP1) inflammasome and secretion of interleukin 1-beta (IL-1β) [62]. In a recent study [63], our laboratory has shown, using murine macrophages, that probenecid treatment inhibits the Nod-like Receptor Protein 3 (NLRP3) inflammasome and associated MAPK signaling pathways. In these studies, probenecid treatment blocked JNK and ERK signaling without affecting p38 MAPK, suppressing NLRP3 inflammasome activation. Moreover, while probenecid does not alter NFκB-directed protein expression, it effectively inhibits outputs of the NLRP3 inflammasome, such as IL-1β production and pyroptosis.

## 6. Interconnection and Communication Between MAPKs

There is continuous communication in a cell as MAPK networks activate genes directly or indirectly. MAPKs form an interconnected intracellular signal transduction pathway to relay, amplify, and integrate signals from diverse stimuli and elicit an appropriate physiological response. The organization of the MAPK signaling cascade is complex, with many interacting pathways and crosstalk. An example is ERK phosphorylation, which affects downstream kinases in the cytoplasm, cell membrane, and nucleus, thus diversifying the signaling cascade. ERK has >160 downstream target molecules [64] that may phosphorylate numerous transcription factors (TFs) that respond to MAPK activation, bind to particular DNA sequences, and regulate gene expression. Pharmacological MAPK inhibitors have helped determine the MAPK signaling pathways and their biological responses to extracellular signals. Using specific antibodies that can distinguish a protein’s phosphorylation status, MAPK activity can be evaluated by quantifying the phosphorylation of MAPK proteins. MAPK inhibitors can help decipher the crosstalk between JNK, ERK, p38, and other downstream kinases, as MAPKs can have a dual role in pro- and anti-apoptotic activities [65,66]. This dual role is partially explained by the diversity of targets activated through different stimuli or by the molecular context where a specific stimulus acts.

As noted, MAPK signaling pathways control proinflammatory responses and are involved in disease pathogenesis. TFs promoting inducible host protein expression may cause over-exuberant cytokine responses, leading to disease. Proinflammatory mediators can increase systemic inflammation while, at the same time, anti-inflammatory cytokines (e.g., interleukin-10, IL-10, and transforming growth factor-beta, TGFβ) are produced in response to proinflammatory signals and down-regulate immune responsiveness. Several proinflammatory cytokines that include IL-1β, TNFα, and IL-6 activate several kinases and induce the activation of stress-activated protein kinase (SAPK), which is part of a signal transduction cascade related to, but distinct from, the MAPK pathway [40]. MAPK regulation is necessary to control inflammation, such as inflammasome activation [67].

Inflammasomes are cytoplasmic innate immune defense complexes and key protectors of cells from pathogens [68]. Inflammasomes typically utilize caspase-1 as their effector protein; however, ‘non-canonical’ inflammasomes are also described, which activate other caspases [69]. NLRP3 inflammasomes may promote and contribute to disease severity during virus infection. Thus, the inflammatory response must be controlled to prevent bystander damage and excessive responses. The p38 MAPK signaling has an essential role in regulating inflammation. Specifically, p38 MAPK is a negative regulator of NLRP3 inflammasome activation [67]. MAPK regulates intracellular Ca^2+^ to reduce pyroptotic cell death—a pro-survival function of p38 MAPK. Proinflammatory cytokines, especially IL-1β, power the inflammatory cascade, although other inflammasomes have been identified, including NLR family CARD domain-containing protein 4 (NLRC4), NLRP1, NLRP6, and others. Studies indicate that the activation of the TLR-JNK axis is needed to activate the NLRP3 inflammasome [70]. JNK can directly phosphorylate NLRP3 [71], a key step in its activation and a crucial priming event for NLRP3 inflammasome assembly and activation. There is evidence that probenecid treatment inhibits NLRP3 inflammasome activation, MAPK signaling, and functional inflammasome outputs, consistent with probenecid’s anti-inflammatory mechanism of action [58,60,72,73]. Additionally, studies have shown that different MAPK pathways (e.g., p38 MAPK) can directly regulate the activation of inflammasomes by phosphorylating key components of the complex and influencing their assembly and activity [74,75]. Given the proinflammatory nature of inflammasomes, it is not surprising that both positive and negative MAPK regulatory mechanisms affect their function.

## 7. Conclusions and Future Direction

The regulation of MAPK pathways is essential for diverse cellular processes such as growth, differentiation, and apoptosis. These pathways are tightly controlled through phosphorylation, feedback loops, and degradation. The MAPK response to virus infection can be modified by probenecid treatment, which inhibits JNK and ERK phosphorylation to preclude virus replication and aspects of inflammation. This review focuses on early MAPK signaling initiated through PRR/TLR activation and viral (RSV) recognition. The review discusses key MAPK components crucial for immunity and preventing inflammation and probenecid’s broad antiviral and anti-inflammatory effects in modifying MAPK responses and virus replication. While viruses like RSV have evolved strategies to evade immunity, the potential for therapeutic approaches such as probenecid treatment to target MAPK pathways to inhibit virus replication and inflammation is a promising area for future research.

A better understanding of MAPK regulatory pathways is needed for viruses that commandeer the host cell for replication, and determining whether these dependencies are virus strain, type, or variant dependent will be essential to controlling replication, transmission, and disease. As viruses engage the ERK/JNK/p38 pathways in their replication, determining the preference in MAPK pathway usage could help direct antiviral strategies and target virus prevention and treatment. Additionally, MAPK inhibitors used in standalone efficacy studies to impair virus replication may demonstrate synergistic effects when combined with other MAPK antiviral agents. Thus, the development of MAPK inhibitors holds significant importance in combating respiratory viral infections.

## Figures and Tables

**Table 1:** The representative IC50 values for several influenza variants are shown for probenecid treatment.

Variant	Viral Strain	Cell Line	IC_50_ µM
H1N1	A/WSN/33	A549	0.00013
H3N2	A/HKx31	A549	0.03
H5N1	A/Vietnam/04	A549	0.003
H7N9	A/Anhui/1/2013	A549	0.016

## References

[1] Farzi R., Pirbonyeh N., Kadivar M.R., Moattari A. (2024). Prevalence of Influenza Viruses A and B, Adenovirus, Respiratory Syncytial Virus, and Human Metapneumonia Viruses among Children with Acute Respiratory Tract Infection. Adv. Virol..

[2] Marchi S., Fallani E., Salvatore M., Montomoli E., Trombetta C.M. (2023). The burden of influenza and the role of influenza vaccination in adults aged 50-64 years: A summary of available evidence. Hum. Vaccines Immunother..

[3] Maggi S., Launay O., Dawson R. (2025). Respiratory Virus Vaccines: Pathways to Recommendations and Enhanced Coverage for At-Risk Populations. Infect. Dis. Ther..

[4] Uyeki T.M. (2020). High-risk Groups for Influenza Complications. JAMA.

[5] Watson A., Wilkinson T.M.A. (2021). Respiratory viral infections in the elderly. Ther. Adv. Respir. Dis..

[6] Hemming V.G. (1994). Viral respiratory diseases in children: Classification, etiology, epidemiology, and risk factors. J. Pediatr..

[7] Walsh E.E. (2017). Respiratory Syncytial Virus Infection: An Illness for All Ages. Clin. Chest Med..

[8] Kaler J., Hussain A., Patel K., Hernandez T., Ray S. (2023). Respiratory Syncytial Virus: A Comprehensive Review of Transmission, Pathophysiology, and Manifestation. Cureus.

[9] Jha A., Jarvis H., Fraser C., Openshaw P.J.M., Hui D.S., Rossi G.A., Johnston S.L. (2016). Respiratory Syncytial Virus. SARS, MERS and Other Viral Lung Infections.

[10] Collins P.L., Fearns R., Graham B.S. (2013). Respiratory syncytial virus: Virology, reverse genetics, and pathogenesis of disease. Curr. Top. Microbiol. Immunol..

[11] Halajian E.A., LeBlanc E.V., Gee K., Colpitts C.C. (2022). Activation of TLR4 by viral glycoproteins: A double-edged sword?. Front. Microbiol..

[12] Chirkova T., Boyoglu-Barnum S., Gaston K.A., Malik F.M., Trau S.P., Oomens A.G., Anderson L.J. (2013). Respiratory syncytial virus G protein CX3C motif impairs human airway epithelial and immune cell responses. J. Virol..

[13] Murawski M.R., Bowen G.N., Cerny A.M., Anderson L.J., Haynes L.M., Tripp R.A., Kurt-Jones E.A., Finberg R.W. (2009). Respiratory syncytial virus activates innate immunity through Toll-like receptor 2. J. Virol..

[14] Bakre A.A., Harcourt J.L., Haynes L.M., Anderson L.J., Tripp R.A. (2017). The Central Conserved Region (CCR) of Respiratory Syncytial Virus (RSV) G Protein Modulates Host miRNA Expression and Alters the Cellular Response to Infection. Vaccines.

[15] Monick M.M., Yarovinsky T.O., Powers L.S., Butler N.S., Carter A.B., Gudmundsson G., Hunninghake G.W. (2003). Respiratory syncytial virus up-regulates TLR4 and sensitizes airway epithelial cells to endotoxin. J. Biol. Chem..

[16] Faist A., Schloer S., Mecate-Zambrano A., Janowski J., Schreiber A., Boergeling Y., Conrad B.C.G., Kumar S., Toebben L., Schughart K. (2023). Inhibition of p38 signaling curtails the SARS-CoV-2 induced inflammatory response but retains the IFN-dependent antiviral defense of the lung epithelial barrier. Antiviral Res..

[17] Borgeling Y., Schmolke M., Viemann D., Nordhoff C., Roth J., Ludwig S. (2014). Inhibition of p38 mitogen-activated protein kinase impairs influenza virus-induced primary and secondary host gene responses and protects mice from lethal H5N1 infection. J. Biol. Chem..

[18] Wang L., Xia Z., Tang W., Sun Y., Wu Y., Kwok H.F., Sun F., Cao Z. (2022). p38 activation and viral infection. Expert. Rev. Mol. Med..

[19] Choi M.S., Heo J., Yi C.M., Ban J., Lee N.J., Lee N.R., Kim S.W., Kim N.J., Inn K.S. (2016). A novel p38 mitogen activated protein kinase (MAPK) specific inhibitor suppresses respiratory syncytial virus and influenza A virus replication by inhibiting virus-induced p38 MAPK activation. Biochem. Biophys. Res. Commun..

[20] Jobe F., Simpson J., Hawes P., Guzman E., Bailey D. (2020). Respiratory Syncytial Virus Sequesters NF-kappaB Subunit p65 to Cytoplasmic Inclusion Bodies To Inhibit Innate Immune Signaling. J. Virol..

[21] Preugschas H.F., Hrincius E.R., Mewis C., Tran G.V.Q., Ludwig S., Ehrhardt C. (2019). Late activation of the Raf/MEK/ERK pathway is required for translocation of the respiratory syncytial virus F protein to the plasma membrane and efficient viral replication. Cell Microbiol..

[22] Rixon H.W.M., Brown G., Murray J.T., Sugrue R.J. (2005). The respiratory syncytial virus small hydrophobic protein is phosphorylated via a mitogen-activated protein kinase p38-dependent tyrosine kinase activity during virus infection. J. Gen. Virol..

[23] Sun Y., Lopez C.B. (2017). The innate immune response to RSV: Advances in our understanding of critical viral and host factors. Vaccine.

[24] Osterholm M.T., Moore K.A., Kelley N.S., Brosseau L.M., Wong G., Murphy F.A., Peters C.J., LeDuc J.W., Russell P.K., Van Herp M. (2015). Transmission of Ebola viruses: What we know and what we do not know. mBio.

[25] He F., Melen K., Maljanen S., Lundberg R., Jiang M., Osterlund P., Kakkola L., Julkunen I. (2017). Ebolavirus protein VP24 interferes with innate immune responses by inhibiting interferon-lambda1 gene expression. Virology.

[26] Furuyama W., Shifflett K., Feldmann H., Marzi A. (2021). The Ebola virus soluble glycoprotein contributes to viral pathogenesis by activating the MAP kinase signaling pathway. PLoS Pathog..

[27] Thompson M.R., Kaminski J.J., Kurt-Jones E.A., Fitzgerald K.A. (2011). Pattern recognition receptors and the innate immune response to viral infection. Viruses.

[28] Li D., Wu M. (2021). Pattern recognition receptors in health and diseases. Signal Transduct. Target. Ther..

[29] Jagodzik P., Tajdel-Zielinska M., Ciesla A., Marczak M., Ludwikow A. (2018). Mitogen-Activated Protein Kinase Cascades in Plant Hormone Signaling. Front. Plant Sci..

[30] Morrison D.K. (2012). MAP kinase pathways. Cold Spring Harb. Perspect. Biol..

[31] Peterson A.F., Ingram K., Huang E.J., Parksong J., McKenney C., Bever G.S., Regot S. (2022). Systematic analysis of the MAPK signaling network reveals MAP3K-driven control of cell fate. Cell Syst..

[32] Kumar R., Khandelwal N., Thachamvally R., Tripathi B.N., Barua S., Kashyap S.K., Maherchandani S., Kumar N. (2018). Role of MAPK/MNK1 signaling in virus replication. Virus Res..

[33] Hirasawa K., Kim A., Han H.S., Han J., Jun H.S., Yoon J.W. (2003). Effect of p38 mitogen-activated protein kinase on the replication of encephalomyocarditis virus. J. Virol..

[34] Sharma N.R., Mani P., Nandwani N., Mishra R., Rana A., Sarkar D.P. (2010). Reciprocal regulation of AKT and MAP kinase dictates virus-host cell fusion. J. Virol..

[35] Cargnello M., Roux P.P. (2011). Activation and function of the MAPKs and their substrates, the MAPK-activated protein kinases. Microbiol. Mol. Biol. Rev..

[36] Kim E.K., Choi E.J. (2010). Pathological roles of MAPK signaling pathways in human diseases. Biochim. Biophys. Acta.

[37] Mercer B.A., D’Armiento J.M. (2006). Emerging role of MAP kinase pathways as therapeutic targets in COPD. Int. J. Chron. Obstruct Pulmon Dis..

[38] Nakano R., Nakayama T., Sugiya H. (2020). Biological Properties of JNK3 and Its Function in Neurons, Astrocytes, Pancreatic beta-Cells and Cardiovascular Cells. Cells.

[39] Ventura J.J., Kennedy N.J., Flavell R.A., Davis R.J. (2004). JNK regulates autocrine expression of TGF-beta1. Mol. Cell.

[40] Cuenda A., Rousseau S. (2007). p38 MAP-kinases pathway regulation, function and role in human diseases. Biochim. Biophys. Acta.

[41] Yokota S., Okabayashi T., Fujii N. (2010). The battle between virus and host: Modulation of Toll-like receptor signaling pathways by virus infection. Mediators Inflamm..

[42] Liu Y., Luo Z. (2024). Repurposing Anticancer Drugs Targeting the MAPK/ERK Signaling Pathway for the Treatment of Respiratory Virus Infections. Int. J. Mol. Sci..

[43] Tripp R.A., Martin D.E. (2022). Repurposing Probenecid to Inhibit SARS-CoV-2, Influenza Virus, and Respiratory Syncytial Virus (RSV) Replication. Viruses.

[44] Widmann C., Gibson S., Jarpe M.B., Johnson G.L. (1999). Mitogen-activated protein kinase: Conservation of a three-kinase module from yeast to human. Physiol. Rev..

[45] Cheng Y., Tian H. (2017). Current Development Status of MEK Inhibitors. Molecules.

[46] Crotty E.E., Sato A.A., Abdelbaki M.S. (2025). Integrating MAPK pathway inhibition into standard-of-care therapy for pediatric low-grade glioma. Front. Oncol..

[47] Ludwig S., Pleschka S., Planz O. (2023). MEK inhibitors as novel host-targeted antivirals with a dual-benefit mode of action against hyperinflammatory respiratory viral diseases. Curr. Opin. Virol..

[48] Cunningham R.F., Israili Z.H., Dayton P.G. (1981). Clinical pharmacokinetics of probenecid. Clin. Pharmacokinet..

[49] Martin D.E., Pandey N., Chavda P., Singh G., Sutariya R., Sancilio F., Tripp R.A. (2023). Oral Probenecid for Nonhospitalized Adults with Symptomatic Mild-to-Moderate COVID-19. Viruses.

[50] Probenecid (2012). LiverTox: Clinical and Research Information on Drug-Induced Liver Injury.

[51] Clark R.S.B., Empey P.E., Kochanek P.M., Bell M.J. (2023). N-Acetylcysteine and Probenecid Adjuvant Therapy for Traumatic Brain Injury. Neurotherapeutics.

[52] Bergeron H.C., Crabtree J., Nagy T., Martin D.E., Tripp R.A. (2024). Probenecid Inhibits Human Metapneumovirus (HMPV) Replication In Vitro and in BALB/c Mice. Viruses.

[53] Murray J., Martin D.E., Hosking S., Orr-Burks N., Hogan R.J., Tripp R.A. (2024). Probenecid Inhibits Influenza A(H5N1) and A(H7N9) Viruses In Vitro and in Mice. Viruses.

[54] Murray J., Bergeron H.C., Jones L.P., Reener Z.B., Martin D.E., Sancilio F.D., Tripp R.A. (2022). Probenecid Inhibits Respiratory Syncytial Virus (RSV) Replication. Viruses.

[55] Murray J., Hogan R.J., Martin D.E., Blahunka K., Sancilio F.D., Balyan R., Lovern M., Still R., Tripp R.A. (2021). Probenecid inhibits SARS-CoV-2 replication in vivo and in vitro. Sci. Rep..

[56] Perwitasari O., Yan X., Johnson S., White C., Brooks P., Tompkins S.M., Tripp R.A. (2013). Targeting organic anion transporter 3 with probenecid as a novel anti-influenza a virus strategy. Antimicrob. Agents Chemother..

[57] Cheng M.H., Kim S.J. (2020). Inhibitory Effect of Probenecid on Osteoclast Formation via JNK, ROS and COX-2. Biomol. Ther..

[58] Jones L.P., Bergeron H.C., Martin D.E., Murray J., Sancilio F.D., Tripp R.A. (2024). Probenecid Inhibits Extracellular Signal-Regulated Kinase and c-Jun N-Terminal Kinase Mitogen-Activated Protein Kinase Pathways in Regulating Respiratory Syncytial Virus Response. Int. J. Mol. Sci..

[59] Wonnenberg B., Tschernig T., Voss M., Bischoff M., Meier C., Schirmer S.H., Langer F., Bals R., Beisswenger C. (2014). Probenecid reduces infection and inflammation in acute Pseudomonas aeruginosa pneumonia. Int. J. Med. Microbiol..

[60] Zheng Y., Tang W., Zeng H., Peng Y., Yu X., Yan F., Cao S. (2022). Probenecid-Blocked Pannexin-1 Channel Protects Against Early Brain Injury via Inhibiting Neuronal AIM2 Inflammasome Activation After Subarachnoid Hemorrhage. Front. Neurol..

[61] Rosli S., Kirby F.J., Lawlor K.E., Rainczuk K., Drummond G.R., Mansell A., Tate M.D. (2019). Repurposing drugs targeting the P2X7 receptor to limit hyperinflammation and disease during influenza virus infection. Br. J. Pharmacol..

[62] Mawhinney L.J., de Rivero Vaccari J.P., Dale G.A., Keane R.W., Bramlett H.M. (2011). Heightened inflammasome activation is linked to age-related cognitive impairment in Fischer 344 rats. BMC Neurosci..

[63] Jones L.P., Martin D.E., Murray J., Sancilio F., Tripp R.A. (2025). Probenecid Inhibits NLRP3 Inflammasome Activity and Mitogen-Activated Protein Kinases (MAPKs). Biomolecules.

[64] McCubrey J.A., Steelman L.S., Chappell W.H., Abrams S.L., Wong E.W., Chang F., Lehmann B., Terrian D.M., Milella M., Tafuri A. (2007). Roles of the Raf/MEK/ERK pathway in cell growth, malignant transformation and drug resistance. Biochim. Biophys. Acta.

[65] Yue J., Lopez J.M. (2020). Understanding MAPK Signaling Pathways in Apoptosis. Int. J. Mol. Sci..

[66] Junttila M.R., Li S.P., Westermarck J. (2008). Phosphatase-mediated crosstalk between MAPK signaling pathways in the regulation of cell survival. FASEB J..

[67] Shin J.N., Rao L., Sha Y., Abdel Fattah E., Hyser J., Eissa N.T. (2021). p38 MAPK Activity Is Required to Prevent Hyperactivation of NLRP3 Inflammasome. J. Immunol..

[68] Yao J., Sterling K., Wang Z., Zhang Y., Song W. (2024). The role of inflammasomes in human diseases and their potential as therapeutic targets. Signal Transduct. Target. Ther..

[69] Downs K.P., Nguyen H., Dorfleutner A., Stehlik C. (2020). An overview of the non-canonical inflammasome. Mol. Aspects Med..

[70] Zhan X., Li Q., Xu G., Xiao X., Bai Z. (2022). The mechanism of NLRP3 inflammasome activation and its pharmacological inhibitors. Front. Immunol..

[71] Song N., Liu Z.S., Xue W., Bai Z.F., Wang Q.Y., Dai J., Liu X., Huang Y.J., Cai H., Zhan X.Y. (2017). NLRP3 Phosphorylation Is an Essential Priming Event for Inflammasome Activation. Mol. Cell.

[72] Garcia-Rodriguez C., Mujica P., Illanes-Gonzalez J., Lopez A., Vargas C., Saez J.C., Gonzalez-Jamett A., Ardiles A.O. (2023). Probenecid, an Old Drug with Potential New Uses for Central Nervous System Disorders and Neuroinflammation. Biomedicines.

[73] Bhaskaracharya A., Dao-Ung P., Jalilian I., Spildrejorde M., Skarratt K.K., Fuller S.J., Sluyter R., Stokes L. (2014). Probenecid blocks human P2X7 receptor-induced dye uptake via a pannexin-1 independent mechanism. PLoS ONE.

[74] Garcia-Hernandez L., Garcia-Ortega M.B., Ruiz-Alcala G., Carrillo E., Marchal J.A., Garcia M.A. (2021). The p38 MAPK Components and Modulators as Biomarkers and Molecular Targets in Cancer. Int. J. Mol. Sci..

[75] Ng G.Y.Q., Loh Z.W., Fann D.Y., Mallilankaraman K., Arumugam T.V., Hande M.P. (2024). Role of Mitogen-Activated Protein (MAP) Kinase Pathways in Metabolic Diseases. Genome Integr..

